# Outstanding Reviewers for *RSC Chemical Biology* in 2021

**DOI:** 10.1039/d2cb90013k

**Published:** 2022-07-14

## Abstract

We would like to take this opportunity to highlight the Outstanding Reviewers for *RSC Chemical Biology* in 2021, as selected by the editorial team for their significant contribution to the journal.
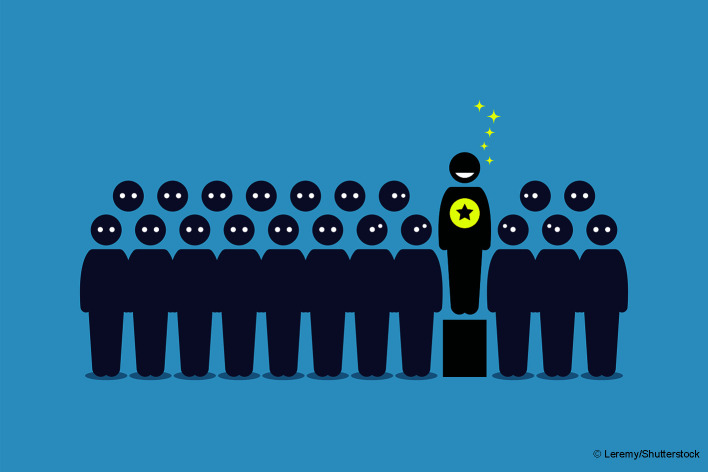

We would like to take this opportunity to thank all of *RSC Chemical Biology’s* reviewers, and in particular highlight the Outstanding Reviewers for the journal in 2021, as selected by the editorial team for their significant contribution to *RSC Chemical Biology*. We announce our Outstanding Reviewers annually and each receives a certificate to give recognition for their contribution. The reviewers have been chosen based on the number, timeliness and quality of the reports completed over the last 12 months.

 

Dr Marco di Antonio

Imperial College London

ORCID: 0000-0002-7321-1867

 

Professor Yimon Aye

École Polytechnique Fédérale de Lausanne

ORCID: 0000-0002-1256-4159

 

Dr Ashraf Brik

Technion-Israel Institute of Technology

ORCID: 0000-0001-8745-2250

 

Dr Martin Grininger

Goethe University

ORCID: 0000-0002-7269-0667

 

Dr Guenter Krause

University of Cologne

ORCID: 0000-0003-3905-0921

 

Dr Mi Hee Lim

Korea Advanced Institute of Science and Technology

ORCID: 0000-0003-3377-4996

 

We would also like to thank the *RSC Chemical Biology* Editorial Board and Advisory Board and the chemical biology community for their continued support of the journal, as authors, reviewers and readers.

 

Dr Anna Rulka, Executive Editor

Sarah Whitbread, Editorial Production Manager

## Supplementary Material

